# Supplementary figures and images for: Peer review and preprint policies are unclear at most major journals
Source: PLoS One. 2020 Oct 21;15(10):e0239518. doi: 10.1371/journal.pone.0239518 (PMC7577440; doi:10.1371/journal.pone.0239518)

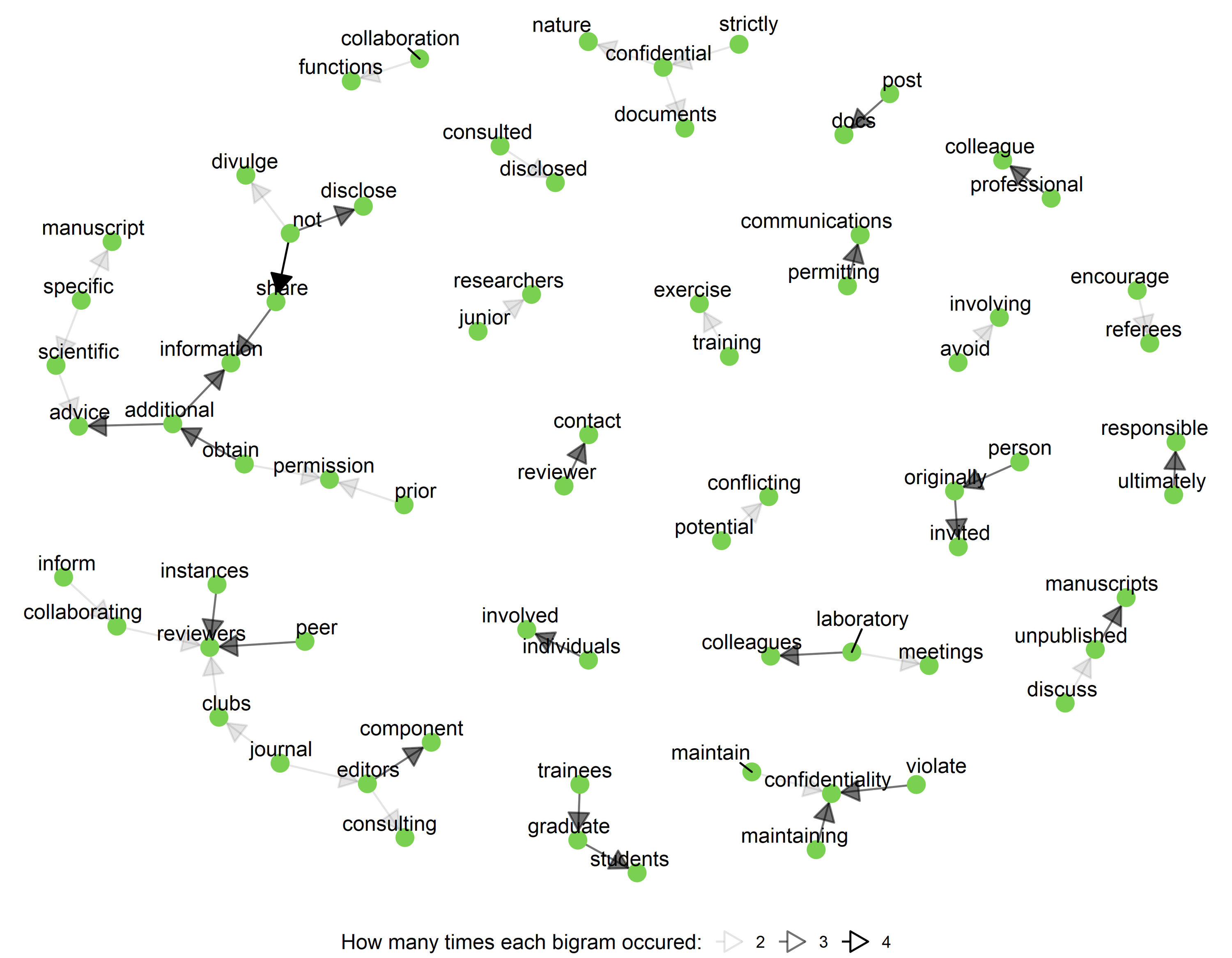

Supplement: S1 Fig — Displayed are bigrams for all terms in the co-reviewing policies, after removal of stop-words (the word “not” was not removed, see the “Methods” section). When creating bigrams, the text is split into pairs of words (for example the sentence "All humans are equal" becomes "All humans", "humans are", "are equal"). The most prominent bigrams were "peer -> review" and "review -> process". To look at the strength of other associations, the term "review" was removed from the figure. The most frequent associations in the figure are depicted by bold arrows. (TIF) [file pone.0239518.s001.tif]
