# Supplementary material for: Peer review and preprint policies are unclear at most major journals
Source: PLoS One. 2020 Oct 21;15(10):e0239518. doi: 10.1371/journal.pone.0239518 (PMC7577440; doi:10.1371/journal.pone.0239518)
Supplement: S1 Table — (PDF) [file pone.0239518.s002.pdf]

S1 Table. Numerical output from Multiple Correspondence analysis.

Variable	mass	inertia	k=1	correlation with dim 1	contribution to dim 1	k=2	correlation with dim 2	contribution to dim 2
Coreview ??	151	48	75	586	33	29	87	86
Coreview ++	49	148	-233	586	103	-90	87	267
Posting preprints ??	72	118	81	305	18	-81	302	320
Posting preprints ++	128	66	-45	305	10	45	302	178
Citing preprints ??	147	52	95	861	52	-19	35	37
Citing preprints ++	53	144	-264	861	143	53	35	102
Revealing reviewer identities to authors ??	115	95	195	728	171	7	1	4
Revealing reviewer identities to authors ++	85	129	-264	728	231	-10	1	6
Peer review ??	62	138	260	853	164	0	0	0
Peer review ++	138	62	-117	853	74	0	0	0
Business, Economics & Management			376	936		-98	64	
Chemical & Materials Sciences			-109	108		313	892	
Engineering & Computer Science			59	70		216	930	
Health & Medical Sciences			-29	67		-107	933	
Humanities, Literature & Arts			169	749		-98	251	
Life Sciences & Earth Sciences			-388	965		74	35	

Physics & Mathematics	-121	757	-68	243
Social Sciences	105	310	-157	690
American Chemical Society	-36	24	233	976
Elsevier	124	1000	0	0
IEEE	206	727	126	273
Royal Society of Chemistry	-158	317	231	683
SAGE	-177	708	-114	292
Springer Nature	-532	915	163	85
Wiley	491	913	151	87
Other publishers	49	77	-170	923
