# Supplementary material for: Peer review and preprint policies are unclear at most major journals
Source: PLoS One. 2020 Oct 21;15(10):e0239518. doi: 10.1371/journal.pone.0239518 (PMC7577440; doi:10.1371/journal.pone.0239518)
Supplement: S2 Table — (PDF) [file pone.0239518.s003.pdf]

S2 Table. Sample phrases for prominent terms in co-review policies

Term	Sample phrase
advice	in some instances, reviewers may feel that it would be helpful to obtain additional *advice* from a colleague.
author	since peer review is confidential, you also must not share information about the review with anyone without permission from the editors and *author*s.
authorization	this means you can't share them with anyone without prior *authorization* from the editor.
authors	since peer review is confidential, you also must not share information about the review with anyone without permission from the editors and *authors*.
collaborate	…reviewers can *collaborate* with trainees (graduate students and post-docs) in the evaluation of manuscripts… however, we ask that reviewers keep the number of collaborators to a minimum and include the identities of all the individuals involved in the "comments to the editors" component of their review.
collaborating	we encourage referees to inform *collaborating* reviewers about appropriate guidelines and ethics for peer review, as outlined in this document.
collaborators	…reviewers can collaborate with trainees (graduate students and post-docs) in the evaluation of manuscripts… however, we ask that reviewers keep the number of *collaborators* to a minimum and include the identities of all the individuals involved in the "comments to the editors" component of their review.
colleague	you should not show the paper to anyone else, including *colleague*s or students, unless you have asked them to write a review, or to help with your review.
colleagues	although referees may consult and seek advice from other researchers or *colleagues*, the referee must ensure that the confidentiality of these materials is preserved.
confidential	annals of internal medicine expects reviewers to handle manuscripts in a *confidential* manner.
confidentiality	… in such cases, we ask that the reviewer contact the editor in advance to ensure that the editor has the opportunity to take additional information into account before permitting communications that have the potential to violate *confidentiality*.
consult	although referees may *consult* and seek advice from other researchers or colleagues, the referee must ensure that the confidentiality of these materials is preserved.
consulted	treat the manuscript as confidential: the manuscript (or its existence) should not be shown to, disclosed to, or discussed with others, except in special cases, where specific scientific advice may be sought; in that event the editor must be informed and the identities of those *consulted* disclosed.
consulting	*consulting* with experts from outside the referee's own laboratory may be acceptable, but please check with the editors before doing so, to avoid involving anyone who may have been excluded by the authors.

Term	Sample phrase
disclose	if you do choose to discuss the manuscript and/or your review with a professional colleague whose input you request as part of your review process, you are responsible for ensuring that they are made fully aware of the confidential nature of the discussion and that they must not *disclose* any information about the manuscript until the article is published.
disclosed	in such instances, the identities of those to be consulted should be *disclosed* to the editor in advance" it is acceptable to consult with laboratory colleagues, but please identify them to the editors.
discuss	we ask referees to treat the review process as strictly confidential, and not to *discuss* the manuscript with anyone not directly involved in the review.
discussed	such documents should neither be disclosed to nor *discussed* with others except, in special cases, when shared in confidence with persons from whom specific expert advice may be sought.
discussion	if you do choose to discuss the manuscript and/or your review with a professional colleague whose input you request as part of your review process, you are responsible for ensuring that they are made fully aware of the confidential nature of the *discussion* and that they must not disclose any information about the manuscript until the article is published.
editor	it is acceptable to consult with laboratory colleagues, but please identify them to the *editor*s.
editors	peer reviewers are required to maintain confidentiality about the manuscripts they review and must not divulge any information about a specific manuscript or its content to any third party without prior permission from the journal *editors*.
identities	however, we ask that reviewers keep the number of collaborators to a minimum and include the *identities* of all the individuals involved in the “comments to the editors” component of their review.
identity	the *identity* of any co-reviewer and any potential conflicting or competing interests they may have must be disclosed when submitting your review.
inform	if you do choose to discuss the manuscript and/or your review with a professional colleague whose input you request as part of your review process, you are responsible for ensuring that they are made fully aware of the confidential nature of the discussion and that they must not disclose any *inform*ation about the manuscript until the article is published.
information	peer reviewers are required to maintain confidentiality about the manuscripts they review and must not divulge any *information* about a specific manuscript or its content to any third party without prior permission from the journal editors.
informed	the manuscript (or its existence) should not be shown to, disclosed to, or discussed with others, except in special cases, where specific scientific advice may be sought; in that event the editor must be *informed* and the identities of those consulted disclosed.

Term	Sample phrase
involve	if you wish to *involve* a specially qualified colleague in the review (or perhaps want to guide a junior colleague in learning how to review), you must contact the editorial office and ask for permission ahead of time.
involved	we ask referees to treat the review process as strictly confidential, and not to discuss the manuscript with anyone not directly *involved* in the review.
involving	consulting with experts from outside the referee's own laboratory may be acceptable, but please check with the editors before doing so, to avoid *involving* anyone who may have been excluded by the authors.
journal	if you wish to have another expert co-review the manuscript with you, you must first obtain permission from the *journal* office.
manuscript	…reviewers can collaborate with trainees (graduate students and post-docs) in the evaluation of *manuscript*s… however, we ask that reviewers keep the number of collaborators to a minimum and include the identities of all the individuals involved in the "comments to the editors" component of their review.
manuscripts	for example, you could share your own *manuscripts* and the reviews you received as an author with your students to facilitate their learning.
not	if you do choose to discuss the manuscript and/or your review with a professional colleague you are responsible for ensuring that they are made fully aware of the confidential nature of the discussion and that they must *not* disclose any information about the manuscript until the article is published.
peer	we encourage referees to inform collaborating reviewers about appropriate guidelines and ethics for *peer* review, as outlined in this document.
permission	if you wish to have another expert co-review the manuscript with you, you must first obtain *permission* from the journal office."
review	a *review*er may request advice from another party, subject to the general principle of confidentiality and notification of the jci.
reviewer	please be sure to contact the handling editor and indicate upon submitting your review that it was completed with a co-*reviewer*.
reviewers	*reviewers* are expected to keep manuscripts confidential.
share	please do not *share* the manuscript with any colleagues without the explicit permission of the editor.
shared	to protect the authors' work as well as your anonymity, communications regarding the manuscript and its parts, including the abstract, may not be *shared* for any reason.
sharing	reviewers should not keep copies of reviewed manuscripts in their personal files and are prohibited from *sharing* copies of the manuscript with others, except with the permission of the editor.
student	we understand that doctoral *student*s need to learn how to write reviews, but there are other methods that can be used.
students	do not have your *students* write your reviews for amr.
