# Supplementary material for: Peer review and preprint policies are unclear at most major journals
Source: PLoS One. 2020 Oct 21;15(10):e0239518. doi: 10.1371/journal.pone.0239518 (PMC7577440; doi:10.1371/journal.pone.0239518)
Supplement: Attachment — Submitted filename: Response to Reviewers.docx [file pone.0239518.s004.docx]

Response to Reviewers

Dear academic editor, dear reviewers,

Thank you for engaging with our work and carefully considering our arguments. We appreciate the insightful comments and questions and have tried to do them justice in a revised version of our manuscript. We are confident this has strengthened the paper and improves re-use of the data we collected.

We respond first to the points raised by the editor, followed by our response to the reviews.

# Editorial issues

*1. Please ensure that your manuscript meets PLOS ONE's style requirements, including those for file naming. The PLOS ONE style templates can be found at*

[*https://journals.plos.org/plosone/s/file?id=wjVg/PLOSOne_formatting_sample_main_body.pdf*](https://journals.plos.org/plosone/s/file?id=wjVg/PLOSOne_formatting_sample_main_body.pdf) *and*

[*https://journals.plos.org/plosone/s/file?id=ba62/PLOSOne_formatting_sample_title_authors_affiliations.pdf*](https://journals.plos.org/plosone/s/file?id=ba62/PLOSOne_formatting_sample_title_authors_affiliations.pdf)

We have updated the formatting according to the templates.

*2. Please include captions for your Supporting Information files at the end of your manuscript, and update any in-text citations to match accordingly. Please see our Supporting Information guidelines for more information: http://journals.plos.org/plosone/s/supporting-information.*

We have included captions for our Supporting Information in the manuscript and revised the Supporting Information according to the guidelines.

*3. Thank you for stating the following in the Competing Interests section:*

*I have read the journal's policy and the authors of this manuscript have the following*

*competing interests:*

*Gary McDowell works at a for-profit that provides consulting services to organizations*

*addressing issues concerning early career researchers. Samantha Hindle is Content*

*Lead at bioRxiv, a preprint server for the biological sciences. Tony Ross-Hellauer is*

*Editor-in-Chief of the journal “Publications” (ISSN 2304-6775).*

*Please confirm that this does not alter your adherence to all PLOS ONE policies on sharing data and materials, by including the following statement: "This does not alter our adherence to PLOS ONE policies on sharing data and materials.” (as detailed online in our guide for authors http://journals.plos.org/plosone/s/competing-interests). If there are restrictions on sharing of data and/or materials, please state these. Please note that we cannot proceed with consideration of your article until this information has been declared.*

We have updated our statement on Competing Interests accordingly. There are no restrictions on sharing of data and/or materials.

*Please include your updated Competing Interests statement in your cover letter; we will change the online submission form on your behalf.*

*Please know it is PLOS ONE policy for corresponding authors to declare, on behalf of all authors, all potential competing interests for the purposes of transparency. PLOS defines a competing interest as anything that interferes with, or could reasonably be perceived as interfering with, the full and objective presentation, peer review, editorial decision-making, or publication of research or non-research articles submitted to one of the journals. Competing interests can be financial or non-financial, professional, or personal. Competing interests can arise in relationship to an organization or another person. Please follow this link to our website for more details on competing interests: http://journals.plos.org/plosone/s/competing-interests*

Thank you for emphasizing this point. To increase transparency, we have updated our Competing Interests statement which now mentions that as junior authors we have a vested interest in improving the publication landscape.

# Reviewer comments

## Reviewer #1

*I enthusiastically recommend publication of the manuscript, which quantifies a problem with the publishing industry that is both widespread and relatively easy to fix. I hope that it will be widely read and that journals that are currently exhibiting the problems it highlights will change their guidance accordingly.*

*The manuscript is well-written and understandable. The methods are detailed and quantitative, and the data are openly shared so that anyone can check the results. The conclusions are justified by the results, and the authors explicitly acknowledge the limitations of the study. In short, it is rigorous, readable, and important.*

*My one substantive concern is that the data shared on Zenodo are poorly organized and poorly annotated, which would likely make their reuse difficult. The HTML files are helpful in this regard and very user-friendly, but it is not clear to me, for example:*

** Where I would find plain-language descriptions of the columns in the first two figures in 01-overview.html. I’m sure that the meaning of “bibjson.publisher” is clear to the authors, but it is not clear to me.*

** Where I would find the raw data (preferably in csv format) for pretty much anything.*

** How I could identify the individual points in part A of h-indices-1.png.*

*There is a ton of data here, presumably everything that was analyzed for this study, and I applaud the authors for sharing it. However, if I were looking for the data underlying a particular analysis, I wouldn’t know where to start. The examples above are just spot-checked; I have not attempted to be thorough, and I’m not suggesting just fixing these. What I am suggesting is overhauling the organization of the data so that an interested reader could easily find the raw data underlying any analysis along with a description of what they mean. I am, in other words, suggesting that the data be organized and structured in accordance with open data best practices, for example with the recommendations of Whitlock 2011 (TREE 26: 61-65* [*https://doi.org/10.1016/j.tree.2010.11.006*](https://doi.org/10.1016/j.tree.2010.11.006)*) (this is just the relevant publication that I’m most familiar with; I’m sure there are more recent, and possibly more detailed, reviews).*

We thank reviewer #1 for their feedback and appreciate that they took the time to look into the data we supplied. We agree that data and code could be organised and presented in a way that better enables data re-use.

Overall, our approach has been to use the `drake` package (<https://books.ropensci.org/drake/>) which streamlines the process of reproducing our analysis. We acknowledge that in doing so, we used a rather complex machinery which does not immediately make apparent all relevant source data to someone unfamiliar with the package. This approach has been partly chosen by the desire to publish the data along with all analytical steps taken, since the final figures (and the data used for plotting) are derived from the original raw data via multiple steps (which can be primitive like fixing typos, but sometimes involve interpretation, like when deciding which categories to collapse, based on cell frequencies and substantive considerations). One could even argue that we barely have any kind of “raw data”, since the data we collected manually is already a processed form of data (the person doing the review chooses a category for a given question like which type of peer review a journal studies). For the purpose of our analysis however, we treat the initial result of our manual landscape scan as “raw data”.

To be absolutely transparent about the process of going from “raw data” to “final figure”, we published (a) the raw data (i.e. data on journals’ policies collected manually) (b) intermediate processed data files (after basic data cleaning procedures) and (c) all code which is necessary to get from (b) to the final figures.

This approach is documented in the README.md, which was expanded considerably in response to the reviewers’ comments. The README points the reader to:

- `plan.R`: This file lays out the analysis steps, detailing which data is used when, which function is used to process which data, etc. Based on the reviewer’s feedback, this file has been improved by explicitly stating which raw data files are used at which step. We also created a static display of the analysis pipeline at `documentation/analysis_pipeline.png`.
- `02-analysis-writeup.html`: this file holds all text and figures on which the paper is based. We improved the README by explicitly mentioning `02-analysis-writeup.Rmd` which holds all code for the analysis.
- Several data files:
  - The original raw data files on GoogleSheets
  - The fact that these have been preserved as .csv and .xlsx
  - Two processed data files upon which all subsequent analysis builds

Documentation of the data has been improved via a range of steps:

- A plain-language file describing the columns of the raw data has been available previously (`var_overview.csv`). This file is now also mentioned in the README.
- Data for all figures has been exported to the directory `data/figures`. Access to the data underlying the figures has in fact been requested by multiple individuals reporting on the study. The files shared contain journal-level data for all figures. This enables both re-creating the figures (by aggregating the data) but also re-analysing the data in different ways.
- The README has been expanded to better guide readers to the relevant data and code files, as explained above. This includes a table of all data files along with a short description of their content and purpose. We have also added a table describing the purpose of all code files relevant to the analysis.

As reviewer #1 rightly points out, we also supply further code and data, which is everything that was done during the analysis. All steps taken are recorded in the git repository (available at <https://github.com/transpose-publishing/landscapeStudy>), which includes an annotated record of all changes (<https://github.com/transpose-publishing/landscapeStudy/commits/master>). The purpose of sharing all these steps is to increase transparency about all steps taken.

The file mentioned specifically by reviewer #1 (`01-overview.html`) was created early in the analysis while exploring the datasets. It includes remarks on observations made and different approaches to visualising the same data and is not intended as a supplemental file which is documented for the reader. Annotating such a file so it can be easily understood would be highly unusual. We would also not want anyone drawing conclusions from these figures, since they are, as stated, preliminary, and have not been vetted for accuracy. We still see merit in sharing these files, should the case arise that some parts of the analysis have to be justified.

## Reviewer #2

*The paper is sound technically but there is repetition. Also rather than present the data which would be new, editorial commentary is mixed in. I have been submitting papers for review for 50+ years. I am also the founding editor of a peer-reviewed journal. The paper has a tone of being authors who are junior who are frustrated with the review process. In many cases, peer review policies are not clear. But an author can contact the journal to clarify issues. It is becoming increasingly difficult to for editors to obtain reviewers. This paper is written from the author's perspective, but this perspective is only one that is important.*

*I take a dim view of co-review. When I sent a paper out for review, I expected the person to whom the review was sent to actually review the paper. Giving the paper to grad students for review was not desirable.*

We thank reviewer #2 for their thorough reading of the manuscript. We have revised the manuscript in several regards responding to all points raised.

- We removed repetition in several places and have removed subjective language from our presentation of the results. We concede that the discussion section contains statements that are subjective in the sense that they comment facts which are undesirable from a junior scholar’s perspective.
- The main criticism of reviewer #2 seems to be that our paper takes the view of junior scholars, whereas for experienced scholars of a given field many of the issues we raise are not of concern. As stated in the abstract and throughout the text, this is exactly our point: Unclear policies make it especially hard for scholars who have not (yet) incorporated the norms and practices of a given field (either because they are junior or because they are new to a field) to navigate the space of scholarly publishing. Deciding on which journals to consider thus becomes increasingly complex, which is inefficient for the process of scholarly communication. It is also an issue for meta-research: tracking the development of certain practices is impeded if there are no or unclear policies on a given subject. We have hence added text throughout the manuscript to emphasise this point.

We respond to all further comments below.

*lines 43-5. there is singular for "graduate student" followed by "their." This happens throughout the paper.*

This is a deliberate choice to avoid using a binary gender. Use of the singular “they” is endorsed by the APA and Merriam-Webster’s Dictionary (see <https://apastyle.apa.org/style-grammar-guidelines/grammar/singular-they> and also <https://www.vox.com/2019/12/13/21011537/they-merriam-webster-pronouns-nonbinary-word-year>). We would prefer to keep it that way. However, if the editor finds it inappropriate, we can also change it to the female form.

*line 46. The pre-print issue is complex. It is possible that there can be no consistent policy. For example, publicizing the paper to journalists may be undesirable. But pre-prints in a professional working paper series may be permissible but this may depend on the working paper series.*

We agree that there might be cases where a consistent policy is not possible. In fact, some of the policies reviewed in our study mention working paper series as permitted forms of prior publications. However, sometimes it is not clear what will count as a prior publication which precludes submission, because this decision is deferred to the editor. Experienced scholars might know how certain journals (and editors) handle certain types of prior publications, but we vouch for this information to be stated clearly on journals’ websites. This would enable less privileged authors and those starting out in a field to assess their options more easily.

Of all the journals in our sample with unclear policies, many actually have no publicly available policy on preprints. This is becoming less of an issue, however, as preprints are becoming more popular which prompts journals to include specific statements on preprints into their author guidelines.

*line 60. I doubt the statement is valid.*

The statement in question is: “Varied and vague policies restrict author choices, and any constraints become more complicated with each additional journal considered.”

We agree that the statement is not correct. Varied and vague policies don’t necessarily restrict author choices, but they inhibit authors' abilities to know and/or understand what their choices are. We have updated the manuscript accordingly.

*line 149-150. In my discipline, there are a few top journals. Their policies are clear to the scholars in the discipline. Admittedly, junior scholars typically want to publish in these journals and are disappointed when their job market papers are not accepted by the top journals.*

We agree that publication policies might be clear to scholars which are highly experienced in their field. As stated above, we have added text in multiple places to indicate the high relevance of clear policies to junior scholars or researchers new to a field.

*line 200. Would the editors not want to control co-review? Co-reviewers may not have the requisite knowledge to review the paper.*

In our view, co-review might be a reasonable practice when it is employed to train junior scholars. However, the main argument in our paper in this regard is that co-reviewing clearly is happening (see ref 18 from our paper) and it would therefore be advisable for journals to have a policy on it.

*Table 1. I have trouble with some of the terms in the left column. Could a phrase be added with the term in italics?*

We added a new column to the table which displays up to three variants for each term that appear in our data. For example, the term “inform” refers among others to the words “information”, “inform” and “informed”. We feel this is the most succinct way of displaying more context about how certain terms appear. We also provide a new supplemental table (S2 Table) where we display one randomly sampled phrase per term variant.

*Line 247. The issue of pre-print is becoming more complex with articles being posted in e.g. Web of Science in advance of publication. Would you consider these papers "published?"*

If we understand the reviewer correctly, they are referring to articles labelled as “early access” or “published ahead of print” (<https://support.clarivate.com/ScientificandAcademicResearch/s/article/Web-of-Science-Core-Collection-Early-Access-articles?language=en_US>). We would consider these papers “published”, since they have been accepted for publication by a journal. The only difference to regular journal articles is the timing of publication. They are type-set by the journal itself, appear on their website, and share any access restrictions the journal might impose. According to the SHERPA/RoMEO definitions, this is the “Published Version” or “Version of Record” (<https://v2.sherpa.ac.uk/romeo/about.html>).

Preprints on the other hand are formatted and posted by the authors. These can be either the submitted version (pre-print) or the accepted version (post-print). These two variants are discussed in our paper.

We have added a sentence in the introduction clarifying this definition.

Kind regards,

Thomas Klebel & Tony Ross-Hellauer
